# Tumoral calcinosis in the lumbar spine secondary to systemic sclerosis: a rare cause of radiculopathy in an adult with advanced disease

**DOI:** 10.1259/bjrcr.20150435

**Published:** 2016-07-28

**Authors:** Afonso Celso Pedrotti Liberato, Lazaro Luiz Faria Do Amaral, Victor Hugo Rocha Marussi

**Affiliations:** Neuroradiology, Beneficencia Portuguesa Hospital, Med Imagem, São Paulo, Brazil

## Abstract

Calcinosis is frequently associated with systemic sclerosis (SSc) and can be located at various sites, although it is most commonly seen in the hands. When it presents around the synovial joints and is associated with a mass-like appearance, it is classically called tumoral calcinosis. Few cases of tumoral calcinosis have been reported in the paraspinal region. They are usually located in the cervical segment and rarely in the lumbar region. Occasionally, they have been associated with nerve root compression and intraspinal extension. We report the case of a 47-year-old female with advanced SSc who presented to our hospital’s radiology department with chronic low back pain and right L5 radiculopathy due to tumoral calcinosis. An initial lumbar spine MRI showed multifocal, low signal, mass-like lesions involving the right paraspinal soft tissues. At the L5–S1 level, one lesion compressed the right L5 exiting nerve root. A CT scan of the lumbar spine performed later demonstrated the calcified nature of the lesions depicted by MRI and evidenced signs of pulmonary fibrosis at the base of the lungs. Further clinical work-up also showed that the patient had Raynaud's phenomenon, oesophageal dysmotility, sclerodactyly, dyspnoea, facial telangiectasias, generalized weakness and arthralgia. The diagnosis of a subtype of SSc, called limited cutaneous SSc, was made. Our case describes the CT and MRI findings of tumoral calcinosis in an unusual location secondary to limited cutaneous SSc. Knowledge of the imaging features of this uncommon manifestation of SSc could potentially increase its prospective diagnosis and hence improve patient management.

## Background

Systemic sclerosis (SSc) is a chronic systemic connective tissue disorder of unknown origin. Middle-aged females are affected more frequently than males. It is characterized by excessive collagen deposition (fibrosis) in the skin and microvasculature, and autoimmune abnormalities. Two overlapping forms of the disease are seen: diffuse SSc and limited cutaneous SSc (lcSSc). The latter is classically associated with calcinosis, Raynaud’s phenomenon, oesophageal dysfunction, sclerodactyly and telangiectasias.^1^

Calcinosis is defined as deposition of calcium salts in tissues. Calcinosis is frequently associated with SSc, especially in the hands and in advanced stages of the disease. It has also been reported to occur in the wrists, forearms, elbows, shoulders, knees, around the iliac crests, over the gluteal regions and the thighs.^2^ It is less commonly seen in the paraspinal soft tissues, particularly in the cervical spine.^3^ Even less frequently, calcinosis can involve the thoracic or the lumbar spine and cause neurological manifestations owing to foraminal and/or intraspinal extension.^4,5^ When calcinosis presents as lobulated periarticular soft tissue masses, usually at the extensor surface of the joints, it is classically known as tumoral calcinosis.^6^ Here, we present a rare case of tumoral calcinosis in the lumbar spine secondary to lcSSc, and highlight its typical imaging findings on both CT and MRI.

## Case report

A 47-year-old female presented to an outside institution with chronic low back pain and right L5 radiculopathy, and an MRI of the lumbar spine was performed (not shown). It showed a mass-like lesion at the right L5–S1 foramen and a diagnosis of hernia or tumour was considered. Over a 1-month period, the patient was managed clinically with analgesics with partial resolution of symptoms.

**Figure 1.**
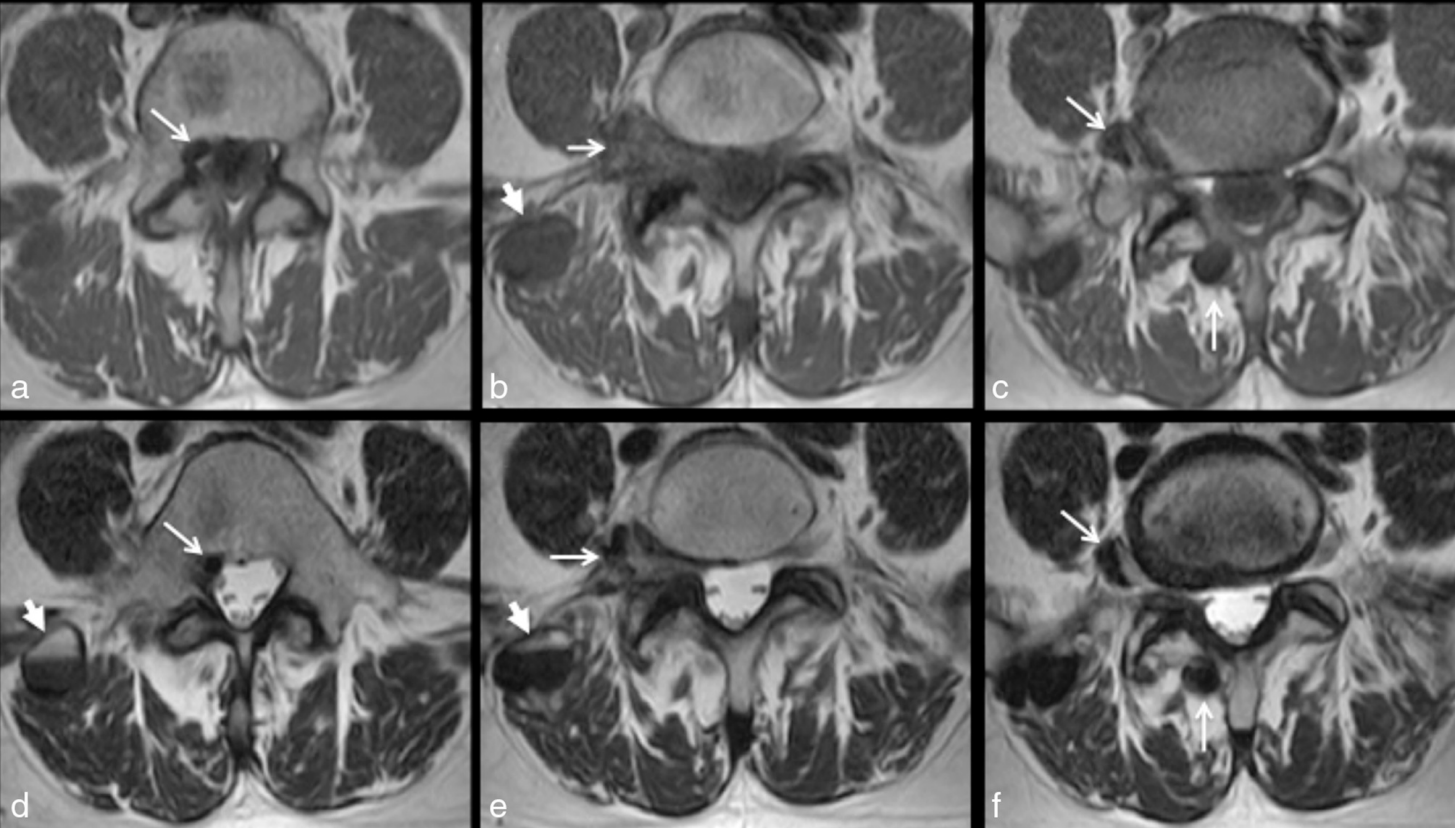
MRI of the lumbar spine. Axial *T*_1_ (a–c) and *T*_2_ weighted (d–f) MRI showing multiple mass-like lesions (white arrows) in the right paraspinal soft tissues. They show low signal intensity compared with adjacent muscles, both on *T*_1_ and *T*_2_ weighted sequences. The right paracentral epidural space (a, d) illustrates a small lesion posteriorly dislocating the L5 descending nerve root. In addition, at the L5–S1 level, foraminal/extraforaminal compression of the right L5 nerve root can be seen (b, c, e, f). The right paraspinal muscle lesion (d, e) demonstrates the sedimentation sign of layered calcium in the lesion (white arrowheads).

**Figure 2.**
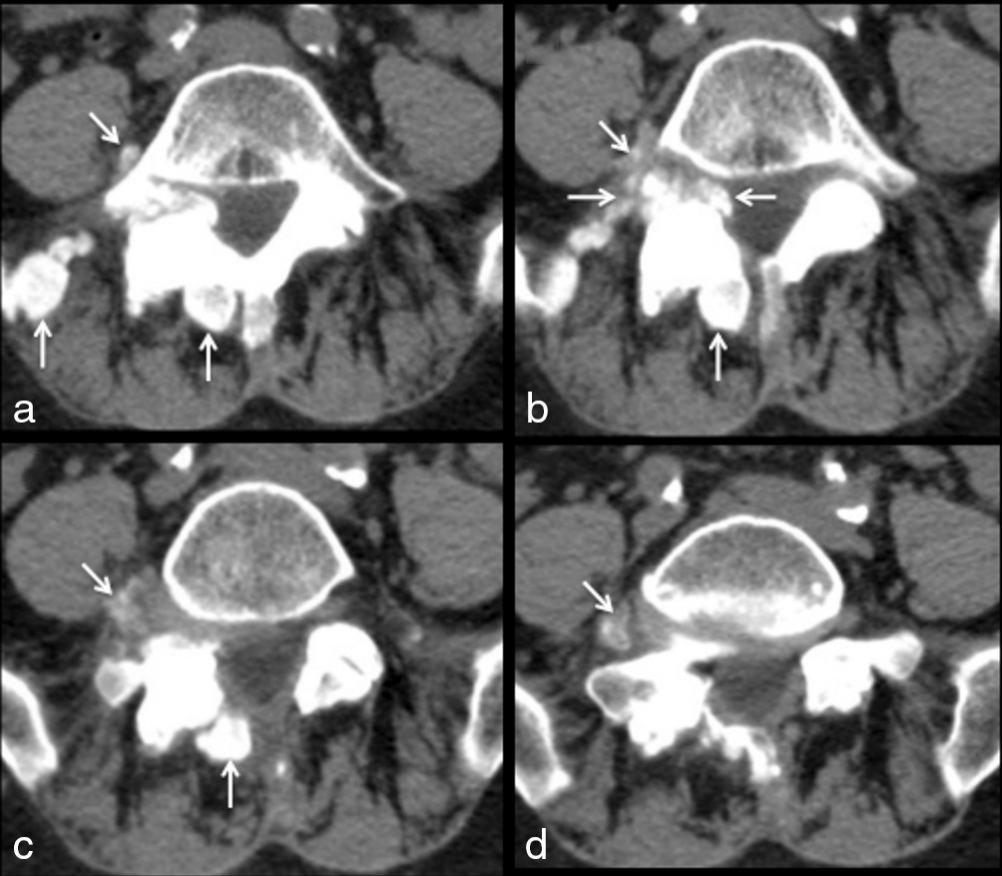
CT scan of the lumbar spine. Axial CT images with soft tissue window (a–d) showing calcified mass-like lesions in the paraspinal soft tissues at the same locations described on MRI (white arrows). The symptomatic lesions involve the right L5–S1 neuroforamen and have extraforaminal extension. The lesion located at the right paracentral epidural space is not well seen on CT owing to its lower attenuation compared with the other lesions.

**Figure 3.**
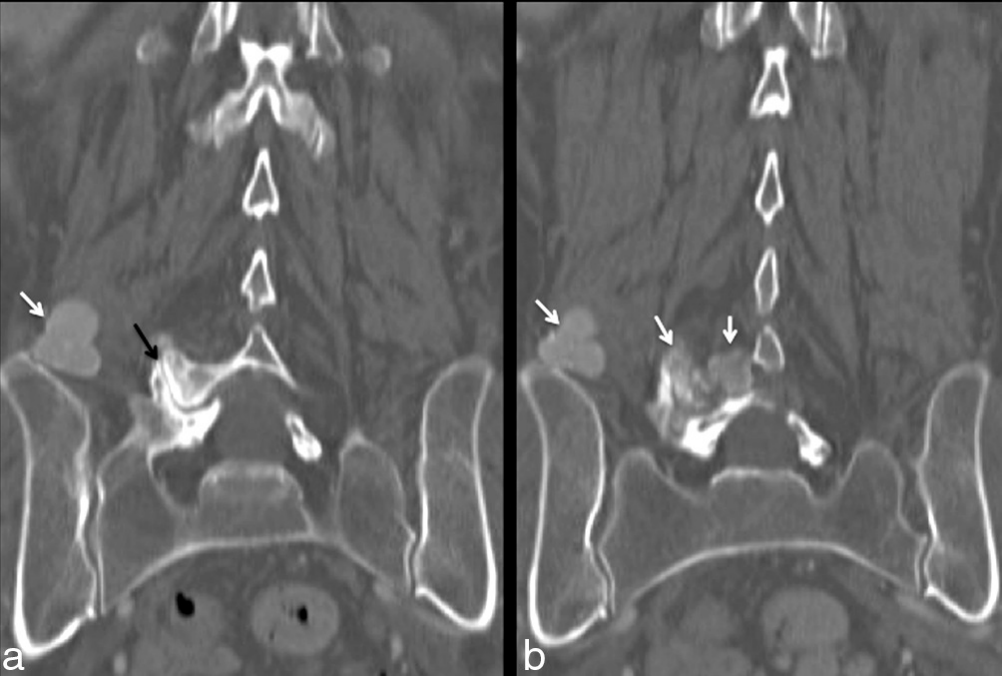
CT scan of the lumbar spine. Coronal reformatted CT images with bone window (a, b) showing calcified mass-like lesions in the right paraspinal soft tissues (white arrows). It also highlights the foraminal lesions centered on the right facet joint (b) that demonstrates severe degenerative joint disease (black arrow).

**Figure 4.**
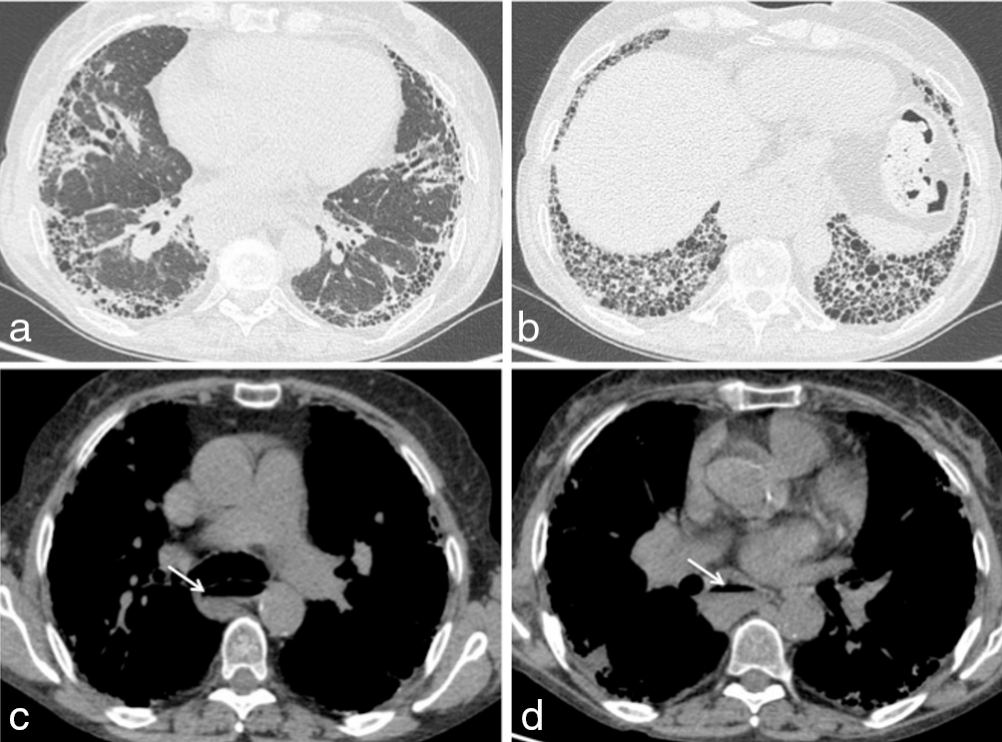
CT scan of the chest. Axial CT images with lung window (a, b) showing signs of bilateral interstitial lung disease with craniocaudal gradient pattern, compatible with pulmonary fibrosis. Axial CT images with soft tissue window (c, d) demonstrating oesophageal dilatation associated with air–fluid level (white arrows).

The patient then presented to the emergency department of our hospital owing to her persistent symptoms and an MRI of the lumbar spine (*T*_1_ and *T*_2_ weighted sequences) was obtained (Figure 1). At this point, no contrast injection was performed as it is not included in the low back pain/radiculopathy investigation protocol in the emergency department. It showed the previously described lesion at the right L5–S1 foramen, as well as other ipsilateral lesions in the posterior paraspinal muscles and the anterior epidural space. Most of the lesions had a mass-like appearance and low signal intensity in all MR sequences. Interestingly, one lesion in the posterior paravertebral muscle showed mixed signal intensity on *T*_2_ weighted sequence, defining a fluid–fluid level. All the lesions were unchanged compared with the outside scan. The radiology team suspected that the lesions were calcified and suggested a contrast-enhanced CT scan of the lumbar spine (Figures 2 and 3) to confirm their initial suspicion and rule out possible soft tissue components associated with the lesions. The CT scan confirmed the calcified nature of the lesions and also showed no contrast enhancement. It also contributed to an additional finding of interstitial lung disease, as seen in a few images at the base of the lungs in the thoracolumbar region. A CT scan of the chest was suggested (Figure 4) and showed signs of pulmonary fibrosis and oesophageal dilatation. At this time, the hypothesis of paraspinal tumoral calcinosis secondary to SSc was suggested.

Additional clinical investigation showed that the patient had a history of dyspnoea, generalized weakness, arthralgia and gastro-oesophageal reflux disease. On physical examination, Raynaud’s phenomenon with a distal phalanx skin ulcer of the left third digit, sclerodactyly and multiple facial telangiectasias were also observed. Laboratory analysis indicated that creatinine, calcium and phosphorus levels were normal. In fact, the patient had an established diagnosis of lcSSc over a period of 15 years. However, this information was not provided to the radiology department, making the diagnosis of lumbar paraspinal tumoral calcinosis secondary to lcSSc challenging.

After a multidisciplinary team meeting, it was initially decided that the patient’s radiculopathy should be managed clinically with non-steroidal and steroidal anti-inflammatory drugs and follow-up MRI of the spine should be performed within 6 months. In case of non-resolution or worsening of the symptoms during the follow-up interval, it was decided that surgical decompression could be considered depending on the patient’s general clinical status.

## Discussion

Paraspinal calcinosis can be related to either the diffuse form of SSc or to lcSSc.^7,8^ Paraspinal calcinosis tends to have a mass-like appearance and is centered around the facet or atlantoaxial joints. The pathophysiology of soft tissue calcification in SSc is still not completely understood, although it is hypothesized that local factors such as degenerative joint disease may play a role in their development.^9^ In addition, other foci of soft tissue calcification can be observed in the adjacent soft tissues, not limited to the vicinity of the joints.^7,10^ In a PubMed database search using the terms calcinosis, SSc/scleroderma and/or spine/spinal/paraspinal, we found 32 cases with imaging descriptions of paraspinal tumoral calcinosis associated with SSc. The majority of the cases involved the cervical spine, followed by the lumbar and thoracic segments.^3,5^ In addition, tumoral calcinosis can also be seen in other parts of the body, especially in juxta-articular locations.^2^ Usually, it shows progressive enlargement over time as well as a tendency to recur if surgically removed.

Tumoral calcinosis has been classified into three groups according to its underlying pathogenesis: primary normophosphataemic, primary hyperphosphataemic and secondary.^11^ The most common is the secondary group, which includes disorders known to promote soft tissue calcification such as renal failure, haemodialysis, sarcoidosis, crystal deposition diseases or collagen vascular disease, as in our report. Unfortunately, many of these different aetiologies of tumoral soft tissue calcinosis cannot reliably be differentiated by imaging as they share similar radiological features. Hence, additional laboratory analysis with serum chemistry levels may be needed to guide the correct diagnosis.^6^

Not uncommonly, tumoral calcinosis has previously been mistaken for neoplasm, degenerative spine disease, infection or even hernia, which delays the correct diagnosis, as in our case.^7,9^ In addition, when other diagnoses are clinically suspected and these calcified lesions are biopsied, they are often non-confirmatory owing to a misinterpretation of the biopsied tissues by pathologists.^3,5,9,12^

Despite this pathology-based classification, some authors believe that the term “tumoral calcinosis” should follow the historical definition. Hence, this term should be strictly used to suggest diseases caused by a hereditary metabolic dysfunction of phosphate regulation associated with massive periarticular calcinosis. In addition, the “secondary” causes of tumoral calcinosis would be better categorized as dystrophic and metabolic (metastatic) calcifications, according to serum chemistry levels (calcium and/or phosphate). Dystrophic calcification results from underlying damaged tissues and includes a wide range of pathologies (*i.e.* connective tissue diseases, infections, trauma, neoplasms, etc.). It occurs in either a localized or generalized distribution and is found in patients with normal serum chemistry levels. On the other hand, metabolic calcification usually results in generalized mineral deposition owing to a disorder of calcium/phosphate metabolism (*i.e.* chronic renal failure, primary hyperparathyroidism, hydroxyapatite deposition disease, etc.).^6^

Paraspinal tumoral calcinosis may be asymptomatic or may present with local pain, discomfort, stiffness, weakness and a decreased range of motion of the neck.^8,10^ In complicated cases, it may present with radiculopathy and/or spinal cord compression syndrome.^4^

Radiography may be sufficient to diagnose tumoral calcinosis lesions, described as amorphous, rounded opacities with a mass-like appearance, although they may not be seen in the early stages. CT is the best examination to detect and locate soft tissue calcified lesions.^2,9^ Usually, they present as large and lobulated masses with homogeneous or heterogeneous density. It may be associated with erosions of the adjacent synovial joints and become the source of instability disorders of the spine.^10^

On MRI, lesions typically show low intensity on *T*_1_ and *T*_2_ weighted images without contrast enhancement.^3,7,12,13^ However, mixed signal intensity and associated peripheral enhancement have been described.^4,14^ MRI is crucial in showing the relationship of the lesion with the neural structures (spinal cord and nerve roots), which may be important in patient management.^3,7^

Occasionally, tumoral calcinosis lesions may show fluid sedimentation level on ultrasound, radiography, CT or MRI. This characteristic finding occurs owing to calcium crystal sedimentation within the dependent portion of the lesion and is recognized as the “sedimentation” sign.^15^ It has been described in both the primary and secondary forms of tumoral calcinosis.^13,16^ On ultrasound examination, tumoral calcinosis lesions may show cystic spaces that contain fluid sedimentation levels, with increased echogenicity of the dependently layering debris.^16^ Similarly, on radiography and CT imaging, a radiodense component within the dependent components of the lesion is seen, while on MRI, the fluid sedimentation level appears as a high signal layered over a low signal on *T*_2_ weighted sequence.^6,13,15,16^ To the best of our knowledge, our case is the first MRI description of sedimentation sign in a patient with SSc.

Several pathologies can present with calcified masses in the soft tissue and involve the paraspinal region, as previously mentioned. Although most tumoral calcinosis aetiologies show similar characteristics on imaging, careful evaluation could potentially raise suspicion for particular entities in the differential diagnosis.^6^

Soft tissue neoplasms, either primary (*i.e.* synovial sarcoma, extraskeletal osteosarcoma, etc.) or secondary (metastasis), should be the first consideration in the differential diagnosis. On imaging evaluation of the spine, multiple lytic or blastic bone lesions, which may or may not be associated with soft tissue components and contrast enhancement, would be helpful findings in favour of a secondary neoplasm aetiology, particularly in a patient with a history of malignant neoplasm and over 40 years of age. In fact, calcified lesions associated with non-calcified soft tissue component and/or contrast enhancement on CT and MRI should promptly raise suspicion for neoplasms over tumoral calcinosis.^6^ On MRI, soft tissue neoplasms may also demonstrate poorly defined and infiltrative margins with mixed signal intensity on *T*_1_ and *T*_2_ weighted sequences owing to the presence of haemorrhage, calcification as well as solid and cystic components. Moreover, a neoplasm may also show bone involvement manifesting as periosteal reaction, superficial erosions or bone destruction, features that are not typical of tumoral calcinosis.^6^ In addition, the sedimentation sign, although non-specific for SSc diagnosis,^13^ may be particularly helpful to distinguish tumoral calcinosis from neoplasm, as in our case.

Myositis ossificans is another entity that may mimic tumoral calcinosis. Although most commonly seen along the large muscle groups of the extremities, it has also been described in the paraspinal region.^17^ Its most typical imaging presentation occurs 6 weeks after onset of the lesion. It presents as a well-circumscribed soft tissue mass with a rim of calcification and no continuity with the adjacent bone. Distinction from tumoral calcinosis can be made by the characteristic myositis ossificans organization into a bone with a distinct cortex and medullary space as well as lack of lobular morphology. Synovial osteochondromatosis is also included in the differential diagnosis of calcified lesions in the paraspinal region, although rarely reported at this location.^18^ Typically, it occurs within a joint, although it may also be present in the periarticular region. On radiography and CT imaging, numerous rounded calcified bodies within an individual joint space associated with an effusion are typically seen. In addition, rings-and-arcs morphology of calcifications may be seen. Together with the intra-articular location of the lesions, these findings help differentiate synovial osteochondromatosis from tumoral calcinosis.^6,18^

There is no current consensus on the treatment of tumoral calcinosis. Pharmacological treatment has been used with variable results in the literature.^5^ Surgical excision of the lesions may be performed when nerve root or spinal cord compression is present. Spinal stabilization can also be performed in selected cases.^4,7^

Our case highlights a rare location and manifestation of tumoral calcinosis secondary to SSc. As the use of MRI as the first imaging tool to investigate radiculopathy increases, it could make the diagnosis of tumoral calcinosis challenging if its typical imaging features are not recognized.

## Learning points

1. Patients with SSc may show paraspinal tumoral calcinosis in the course of the disease, particularly in advanced disease stages, with most being asymptomatic. Occasionally, they may be associated with nerve root compression and intraspinal extension.
2. Tumoral calcinosis should be included in the differential diagnosis of calcified masses in the paraspinal soft tissues, especially in patients with advanced SSc.
3. On MRI, tumoral calcinosis typically shows low intensity on both *T*_1_ and *T*_2_ weighted images without the associated soft tissue component or contrast enhancement.
4. Although a non-specific imaging finding, the “sedimentation sign” due to calcium layering within the calcified lesions may occur, particularly in the *T*_2_ weighted sequence. It can be an additional finding to support a benign aetiology for the differential diagnosis of soft tissue calcifications.

## Consent

Written informed consent was obtained from the patient(s) for publication of this case report, including accompanying images.
